# Rejection Requiring Treatment within the First Year following Heart Transplantation: The UNOS Insight

**DOI:** 10.3390/jpm14010052

**Published:** 2023-12-29

**Authors:** Marco Gemelli, Ilias P. Doulamis, Aspasia Tzani, Athanasios Rempakos, Polydoros Kampaktsis, Paulino Alvarez, Alvise Guariento, Andrew Xanthopoulos, Grigorios Giamouzis, Kyriakos Spiliopoulos, Rabea Asleh, Ernesto Ruiz Duque, Alexandros Briasoulis

**Affiliations:** 1 Department of Cardiac, Thoracic, Vascular and Public Health Sciences, University of Padua, 35122 Padova, Italy; marco.gemelli@studenti.unipd.it (M.G.); alvise.guariento@unipd.it (A.G.); 2 Department of Surgery, Lahey Hospital and Medical Center, Burlington, MA 01805, USA; doulamis.i@gmail.com; 3 Heart and Vascular Center, Brigham and Women’s Hospital, Harvard Medical School, Boston, MA 02115, USA; atzani@bwh.harvard.edu; 4 Medical School of Athens, National and Kapodistrian University of Athens, 157 72 Athens, Greece; 5 Division of Cardiology, Columbia University Irving Medical Center, New York City, NY 10032, USA; pk2644@cumc.columbia.edu; 6 Division of Cardiology, Cleveland Clinic Foundation, Cleveland, OH 44195, USA; alvarep3@ccf.org; 7 Department of Cardiology, University General Hospital of Larissa, 413 34 Larissa, Greece; anxanthopoulos@med.uth.gr (A.X.); grgiamouzis@med.uth.gr (G.G.); 8 Department of Cardiothoracic Surgery, University of Thessaly, 412 23 Larissa, Greece; spiliopoulos@uth.gr; 9 Department of Cardiovascular Diseases, Mayo Clinic, Rochester, MN 55902, USA; rasleh@hadassah.org.il; 10 Heart Institute, Hadassah University Medical Center, Jerusalem 9112001, Israel; 11 Division of Cardiovascular Medicine, Section of Heart Failure and Transplantation, University of Iowa, Iowa City, IA 52242, USA; ernesto-ruizduque@uiowa.edu

**Keywords:** heart transplantation, UNOS Registry, personalized immunosuppression, long-term outcomes

## Abstract

(1) Background: Heart failure is an extremely impactful health issue from both a social and quality-of-life point of view and the rate of patients with this condition is destined to rise in the next few years. Transplantation remains the mainstay of treatment for end-stage heart failure, but a shortage of organs represents a significant problem that prolongs time spent on the waiting list. In view of this, the selection of donor and recipient must be extremely meticulous, considering all factors that could predispose to organ failure. One of the main considerations regarding heart transplants is the risk of graft rejection and the need for immunosuppression therapy to mitigate that risk. In this study, we aimed to assess the characteristics of patients who need immunosuppression treatment for rejection within one year of heart transplantation and its impact on mid-term and long-term mortality. (2) Methods: The United Network for Organ Sharing (UNOS) Registry was queried to identify patients who solely underwent a heart transplant in the US between 2000 and 2021. Patients were divided into two groups according to the need for anti-rejection treatment within one year of heart transplantation. Patients’ characteristics in the two groups were assessed, and 1 year and 10 year mortality rates were compared. (3) Results: A total of 43,763 patients underwent isolated heart transplantation in the study period, and 9946 (22.7%) needed anti-rejection treatment in the first year. Patients who required treatment for rejection within one year after transplant were more frequently younger (49 ± 14 vs. 52 ± 14 years, *p* < 0.001), women (31% vs. 23%, *p* < 0.001), and had a higher CPRA value (14 ± 26 vs. 11 ± 23, *p* < 0.001). Also, the rate of prior cardiac surgery was more than double in this group (27% vs. 12%, *p* < 0.001), while prior LVAD (12% vs. 11%, *p* < 0.001) and IABP (10% vs. 9%, *p* < 0.01) were more frequent in patients who did not receive anti-rejection treatment in the first year. Finally, pre-transplantation creatinine was significantly higher in patients who did not need treatment for rejection in the first year (1.4 vs. 1.3, *p* < 0.01). Most patients who did not require anti-rejection treatment underwent heart transplantation during the new allocation era, while less than half of the patients who required treatment underwent transplantation after the new allocation policy implementation (65% vs. 49%, *p* < 0.001). Patients who needed rejection treatment in the first year had a higher risk of unadjusted 1 year (HR: 2.25; 95% CI: 1.88–2.70; *p* < 0.001), 5 year (HR: 1.69; 95% CI: 1.60–1.79; *p* < 0.001), and 10 year (HR: 1.47; 95% CI: 1.41–1.54, *p* < 0.001) mortality, and this was confirmed at the adjusted analysis at all three time-points. (4) Conclusions: Medical treatment of acute rejection was associated with significantly increased 1 year mortality compared to patients who did not require anti-rejection therapy. The higher risk of mortality was confirmed at a 10 year follow-up. Further studies and newer follow-up data are required to investigate the role of anti-rejection therapy in the heart transplant population.

## 1. Introduction

Heart failure is undeniably one of the most profoundly impactful public health challenges, given its markedly high levels of morbidity and mortality. Moreover, owing to the progressive aging of the population, it has evolved into an escalating social and economic burden. In the United States alone, the affliction touches the lives of more than 5 million individuals, and the prevalence is predicted to surge from 2.4% of the population in 2012 to an anticipated 3% by the year 2030. At present, an alarming figure of over 1 million heart failure patients necessitate hospitalization each year in the United States, with approximately 1 million new cases emerging annually [1,2]. The survival rates following a heart failure diagnosis at 1 year, 5 years, and 10 years stand at 80.8%, 48.2%, and 26.2%, respectively, with heart failure being one of the causes of death for 42.4% of those who died [3]. Notably, a disconcerting 4 to 5% of patients deteriorate to the point of reaching end-stage heart failure, a state characterized by relentless symptoms persisting despite optimal medical intervention, ultimately leading to recurrent hospitalizations and a substantial deterioration in overall functional capacity [4,5]. 

While heart transplantation continues to be the definitive therapeutic intervention for such end-stage heart failure patients, boasting an impressive 1 year survival rate exceeding 90% and a median survival span surpassing 12 years, it is beset by the persistent conundrum of organ scarcity and the resulting protracted waiting lists [4]. In a concerted effort to mitigate waitlist mortality, significant modifications were made to the allocation criteria by the United Network for Organ Sharing (UNOS) in 2018 [6]. These revisions were aimed at accommodating the burgeoning population of patients relying on ventricular assist devices as a bridge to transplantation and, crucially, giving higher priority to individuals with more acute illnesses in anticipation of receiving a heart graft. The 2018 allocation overhaul indeed succeeded in diminishing waitlist mortality, yet it was regrettably associated with an upswing in 1 year post-operative mortality due to the transplantation of patients who were considerably more unwell [7]. 

One of the crucial aspects of a successful organ transplantation is the anti-rejection treatment, for which patients undergo immunosuppressive therapy throughout their lives [4,8]. Despite this, the risk of rejection is always present, and it seems to be particularly deleterious when it happens within the first year after transplantation [9,10]. Rejection after heart transplantation is a known predictor of later post-transplant morbidity and mortality and is one of the major causes of prolonged length of stay, readmissions, and increased costs for the health system [11,12,13]. One of the risks associated with the UNOS policy changes that could potentially lead to an increase in the rate of rejection is the more direct access to heart transplantation for higher-acuity patients. However, Vaidya et al. showed no difference in the early post-transplantation rate of treated rejection and hospitalization for rejection after the new allocation system implementation [9].

Our study aimed to comprehensively evaluate the attributes and traits of patients who experienced graft rejection necessitating therapeutic intervention within the initial year following heart transplantation. Furthermore, we sought to investigate the impact of such rejection episodes on 1 year, 5 years, and 10 years of survival.

## 2. Materials and Methods

### 2.1. Ethical Statement and Study Design

This is a retrospective observational study based on the comprehensive United Network for Organ Sharing (UNOS) Registry, which is the Organ Procurement and Transplantation Network under contract with the United States Department of Health and Human Services. The registry is a prospective data collection initiative that has been meticulously recording information on patients undergoing organ transplantation in the United States (US) since its inception in 1987. This study fully adheres to the ethical principles outlined in the Declaration of Helsinki. Ethical review and approval were waived for this study due to de-identified data from national database with access granted to all heart transplant centers were used. Furthermore, the study strictly adhered to the Strengthening the Reporting of Observational Studies in Epidemiology (STROBE) reporting guidelines. The need for informed consent was waived because this was a secondary analysis of a de-identified dataset. In the UNOS Registry, patients who underwent transplantation prior to the year 2018 were categorized using the “old” UNOS allocation system, which classified them into three distinct classes: 1A, 1B, and 2. In contrast, patients transplanted after 2018 were assessed under the “new” allocation system, which is more comprehensive and encompasses a total of six distinct classes [6]. 

### 2.2. Patients and Outcomes

De-identified patient-level variables for all patients who underwent heart transplantation in the US between 2000 and 2021 were collected from the UNOS Registry. The only exclusion criterion was to be transplanted during pediatric age (<18 years old). Also, combined multi-organ transplantation was not considered. Baseline recipient characteristics included age, gender, race, UNOS status, prior left ventricular assist device (LVAD) implantation, prior cardiac surgery, creatinine, calculated panel reactive antibody (CPRA) value, cardiac output, pulmonary capillary wedge pressure (PCWP), systolic pulmonary artery pressure (sPAP), use of an intra-aortic balloon pump (IABP), and use of extracorporeal membrane oxygenation (ECMO). We also collected donor age, gender, and ischemic time of transplanted hearts. Patients were divided into two groups according to the need for anti-rejection treatment within one year of heart transplantation. Post-operative outcomes were analyzed, and survival data were collected at 1, 5, and 10 years. A subgroup analysis of patients undergoing heart transplantation was performed after the new allocation criteria were met.

### 2.3. Statistical Analysis

Normally distributed continuous variables were expressed as mean ± standard deviation, while categorical variables were expressed as frequencies and percentages. The normality of the data distribution was tested by the Shapiro–Wilk test. Baseline characteristics were compared between groups using the Student’s *t*-test for continuous variables and Pearson’s χ^2^ test for categorical variables. Survival at 1 and 10 years was assessed using the Kaplan–Meier method and compared using the log-rank test. In addition, hazard ratios (HRs) for survival were estimated using a Cox proportional hazards model. Cox regression analysis was adjusted for age, gender, creatinine, UNOS recipient status, prior LVAD, IABP, or ECMO, donor age, donor gender, and ischemic time. All statistical analyses were performed with R Statistical Software, version 4.2.1 (R Foundation for Statistical Computing, Vienna, Austria). A *p*-value of <0.05 was considered to indicate the statistical significance of the differences between the two groups.

## 3. Results

### 3.1. Patient Characteristics

**Figure 1:**
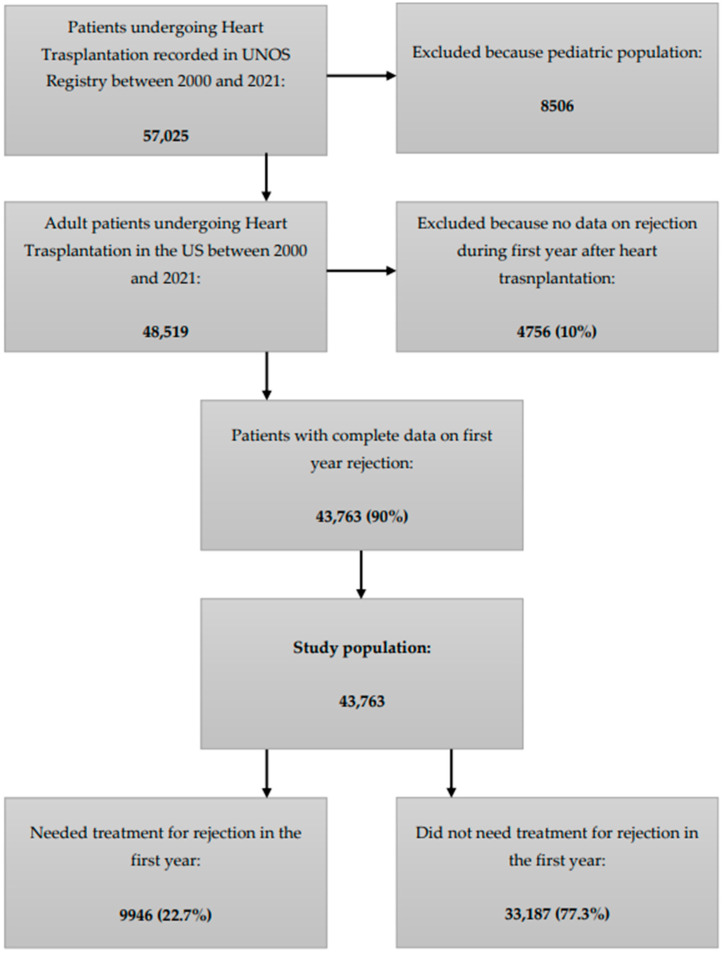
Flowchart representing the characteristics of the UNOS Registry and the study population. UNOS: United Network for Organ Sharing.

According to the UNOS Registry, a total of 57,025 patients underwent isolated heart transplantation in the United States between 2000 and 2021, and 8506 pediatric patients were excluded. Of 48,519 adult patients who underwent heart transplantation, complete data on first year rejection were available for 43,763 (90%) of them, which were included in our analysis and represent the study population. Of this, 9946 (22.7%) had at least one episode of rejection that needed treatment in the first year after transplantation (Figure 1).

In our population, induction therapy was utilized in 46.8% of the population, and anti-thymocyte globulin (ATG) was the most used, followed by IL-2 receptor agonists (IL2RA) (19.5%). Unadjusted analysis for prediction of rejection requiring treatment showed that the use of IL2RA was associated with decreased risk for rejection (IL2RA vs. no induction OR: 0.56; 95% CI: 0.42–0.76; *p* < 0.001), while the use of ATG was not associated with significant changes (ATG vs. no induction OR: 1.07; 95% CI: 0.86–1.33; *p* = 0.572). These observations were persistent after adjusting for risk factors (adjusted analysis for prediction of rejection requiring treatment: IL2RA vs. no induction OR: 0.50; 95% CI: 0.37–0.68; *p* < 0.001; ATG vs. no induction OR: 0.97; 95% CI: 0.77–1.22; *p* = 0.8).

Most of the patients who did not require anti-rejection treatment underwent heart transplantation during the new allocation era, while less than half of the patients who required treatment underwent transplantation after the new allocation policy implementation (65% vs. 49%, *p* < 0.001). Regarding the baseline characteristics, patients who required treatment for rejection within one year after transplantation were younger (49 years vs. 51 years, *p* < 0.001), female (31% vs. 23%, *p* < 0.001), and had a higher CPRA value (14 vs. 11, *p* < 0.001). Also, the rate of prior cardiac surgery was more than double in this group (27% vs. 12%, *p* < 0.001), while prior LVAD (12% vs. 11%, *p* < 0.001), prior IABP (10% vs. 9%, *p* < 0.01), and prior ECMO (2% vs. 1.6%, *p* = 0.007) were statistically more frequent in patients who did not receive anti-rejection treatment in the first year. Finally, pre-transplantation creatinine was significantly higher in patients who did not need treatment for rejection in the first year (1.4 vs. 1.3, *p* < 0.01). Donors’ hearts who experienced rejection in the first year after the transplantation procedure were also statistically younger (31 years vs. 32, *p* < 0.001) and more frequently from deceased women (31% vs. 27%, *p* < 0.001). The detailed patient characteristics of the two groups are represented in Table 1.

### 3.2. Post-Operative Outcomes

Post-operative cerebrovascular accidents (CVA) did not differ between the groups (Adjusted OR: 0.88; 95% CI: 0.73–1.06; *p* = 0.2); however, need for dialysis (Adjusted OR: 0.88; 95% CI: 1.02–1.21; *p* = 0.014) and pacemaker implantation (Adjusted OR: 1.24; 95% CI: 1.08–1.43; *p* = 0.002) were significantly lower in the group that required anti-rejection treatment.

### 3.3. Impact of Rejection Treatment on Survival

**Figure 2:**
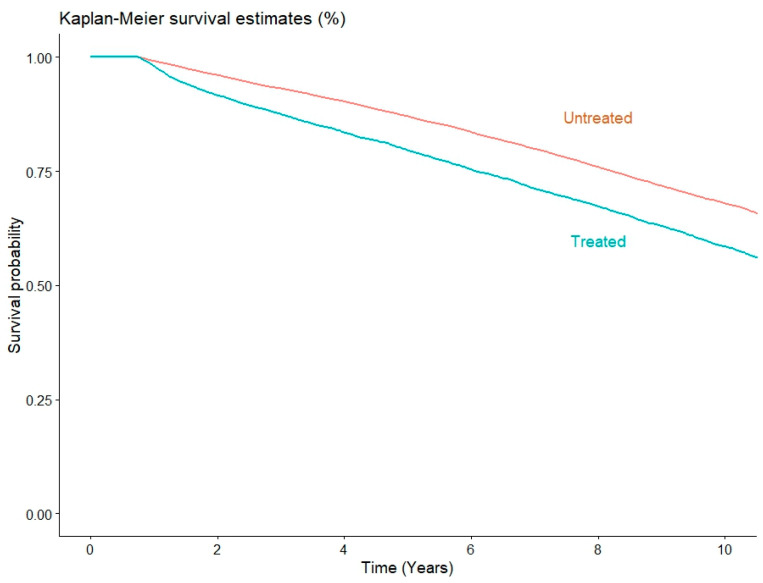
Kaplan–Meier curves represent the long-term survival of patients with rejection who needed treatment in the first year after heart transplantation (red line) and patients without rejection needing treatment during the first year (blue line).

**Figure 3:**
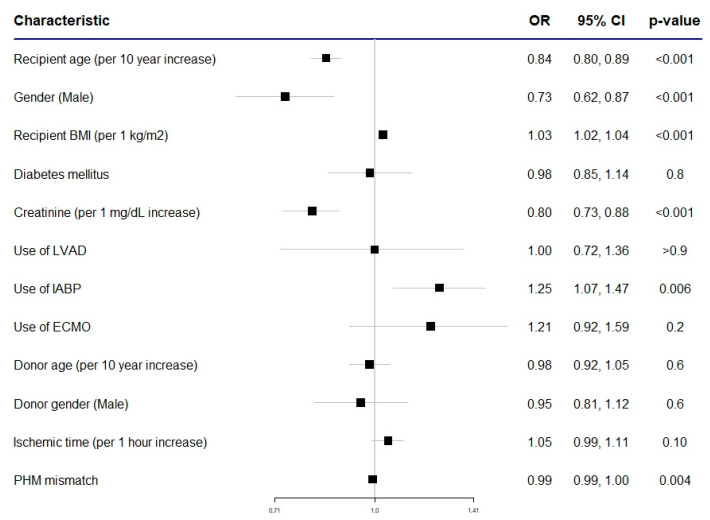
Forest plot representing ORs (odds ratios) for mortality of patients undergoing heart transplantation in the new allocation era. *95%* CI: 95% confidence interval. BMI: body mass index. ECMO: extracorporeal membrane oxygenation. IABP: intra-aortic balloon pump. LVAD: left ventricular assist device. PMH: predicted heart mass.

Survival at 1 year was 98% for patients who needed treatment for rejection in the first year after the transplantation and 99.1% for patients who did not need it, while at 5 years it was 79.6% and 87%, and at 10 years it was 58.5% and 67.9%, respectively. Survival is represented by the Kaplan–Meier curves in Figure 2. Univariate unadjusted Cox regression showed patients who needed treatment for rejection during the first year had a significantly higher risk of mortality at 1 year (HR: 2.25; 95% CI: 1.88–2.70; *p* < 0.001), and this was sustained at 5 years (HR: 1.69; 95% CI: 1.60–1.79; *p* < 0.001) and 10 years (HR: 1.47; 95% CI: 1.41–1.54, *p* < 0.001). Adjusted Cox regression analysis confirmed the results of the unadjusted analysis at 1 year (HR: 2.31; 95% CI: 1.89–2.83, *p* < 0.001), 5 years (HR: 1.73; 95% CI: 1.62–1.84; *p* < 0.001), and 10 years (HR: 1.53; 95% CI: 1.46–1.61; *p* < 0.001). A forest plot summarizing the odds ratio for 1 year mortality in a subgroup analysis of the population that underwent heart transplants with the new allocation system is represented in Figure 3.

## 4. Discussion

The primary findings from this retrospective analysis of data sourced from the UNOS Registry are summarized as follows: (1) over 21 years in the United States, approximately 22.7% of heart transplant recipients necessitated anti-rejection therapy within the initial year following their transplant procedure; (2) individuals requiring anti-rejection therapy tended to be women and of younger age. They also exhibited higher CPRA values and a greater incidence of prior cardiac surgeries. Conversely, the other group, which did not require such therapy, showed a more frequent history of prior IABP and LVAD usage; and (3) the need for anti-rejection treatment during the first year following heart transplantation emerged as an independent risk factor, significantly impacting both the 1 year and 10 year mortality rates among transplant recipients.

The International Society for Heart and Lung Transplantation (ISHLT) Registry is an invaluable repository of data sourced from a vast cohort exceeding 120,000 patients who have undergone heart and lung transplantation procedures on a global scale. This extensive dataset provides a rich reservoir of insights, including the rates of rejection within the first year following transplantation. These rates have exhibited dynamic temporal fluctuations over time, oscillating from a notable 30% in the early 2000s to a more recent, relatively diminished rate of 25%. Concurrently, the prevalence of rejection necessitating therapeutic medical intervention has showcased a downward trend, plummeting from a substantial 23% to a more manageable 13% within the ISHLT’s comprehensive dataset [14,15,16,17]. Within the scope of our study, conducted from 2000 to 2021, we have discerned an overall incidence of treated rejection amounting to 22.7%. Notably, our data has illuminated an intriguing aspect: approximately two-thirds of patients who did not require anti-rejection treatment underwent transplantation after the implementation of new allocation criteria. This begs the question of the impact of these criteria on the post-transplantation landscape. Intriguingly, a study conducted by Vaidya et al. has shed light on this matter. It suggests that despite the adoption of the new UNOS policy, which expanded the pool of medically complex patients eligible for transplantation, there was no apparent increase in the rate of treated rejection [9]. This paradoxical observation hints at the possibility that the UNOS criteria shift favored sicker patients while simultaneously mitigating the effect towards a significant reduction in rejections needing treatment, akin to the trends observed within the ISHLT registry.

Regarding the demographic characteristics of patients who experienced treated rejection, it emerges that these patients are more frequently of the female gender, younger in age, and have a history of prior cardiac surgery. These observations warrant closer examination, beginning with gender. The female sex, recognized as a risk factor for immunosensitization, finds its basis in the immunological memory acquired during pregnancy. Pregnancy constitutes a profound immunological event triggered by the memory of paternal human leukocyte antigens (HLA) encountered during gestation. Such events predispose women to higher sensitization levels and an elevated risk of rejection following organ transplantation [18,19]. Further exploration reveals that, within the context of heart transplantation, females appear to bear a disproportionate burden. They exhibit a higher propensity for episodes of moderate or severe rejection and an increased likelihood of hospitalization for acute rejection (15% vs. 6%, *p* = 0.013). Additionally, women are at greater risk for diminished actuarial survival post-cardiac transplantation [20,21]. Next, we investigated the factor of age, a key determinant of rejection risk. Younger patients, characterized by their robust immune systems and heightened immune responsiveness, face an increased susceptibility to rejection. A study by Wever-Pinzon et al. corroborates this finding, revealing that younger patients face significantly elevated risks of death resulting from acute rejection, cardiac allograft vasculopathy, and graft failure [22]. Examining the donor cohort whose hearts are destined for recipients requiring rejection therapy, we identify a similar trend in terms of age and gender. These donors tend to be younger and, more frequently, female. This underscores the critical importance of meticulous donor–recipient matching in heart transplantation. Research by Jawitz et al. emphasizes this intricate relationship, indicating that while donor–recipient age matching does not significantly impact post-transplant survival, older donors exert a similar negative effect on survival in both older and younger recipients [23]. 

For this reason, it is preferred to favor younger recipients with younger hearts. The significance of gender matching becomes apparent as well. Despite certain studies suggesting that size and weight matching may take precedence over gender, Weiss et al., in their analysis of extensive datasets, provide compelling evidence to the contrary. Their findings suggest that men receiving organs from same-sex donors experience significantly improved short- and long-term survival, while no such survival advantage is observed for women with same-sex donors [24,25,26]. 

Another noteworthy factor is the history of previous cardiac surgery among recipients, which may contribute to heightened rates of treated rejection. Such surgery often involves blood transfusions and extensive contact with synthetic materials during cardiopulmonary bypass, potentially leading to immune sensitization. Extracorporeal circulation during cardiac surgery provokes an inflammatory response, which can lead to the generation of new antibodies. Intriguingly, the literature demonstrates that mechanical circulatory support (MCS) devices, which expose blood to synthetic materials, can elicit a similar immune response [27,28,29,30,31]. Therefore, we expected to have a higher rate of IABP, LVAD, and ECMO in patients who need anti-rejection treatment in the first year after the transplantation procedure. Conversely, our data reveals that MCS devices were marginally more prevalent in the group and did not necessitate treatment for rejection. However, it is important to note that, while statistically significant, this discrepancy may not hold clinical significance owing to the substantial sample size. Sensitization levels also depend on the duration of support with MCS devices [32,33]. 

Finally, as anticipated, the population experiencing treated rejection displays elevated Calculated Panel Reactive Antibody (CPRA) values. These values serve as indicators of the degree of patient immunization, and extensive evidence suggests that elevated CPRA values are linked to an increased risk of rejection and higher mortality rates [29,34]. 

The main finding of our study is that rejection needing treatment during the first year after a heart transplantation was associated with an elevated risk of 1, 5, and 10 year mortality. This finding is particularly intriguing because, while an increased risk of mortality at the 1 year mark is somewhat expected due to the immediate challenges of managing rejection, the persistence of this heightened risk over the very long-term is less conventional. We speculated that certain risk factors or underlying conditions in patients may serve as triggers for multiple episodes of rejection over the years. These recurrent episodes of rejection, even if managed medically, could potentially inflict ongoing damage to the transplanted heart. Over time, this cumulative damage might contribute to the increased mortality risk observed in our study, extending beyond the initial year post-transplantation. Also, patients with a rejection episode during the first year are at increased risk of relapse. This recurrence of rejection could be a contributing factor to the widening gap observed in the Kaplan–Meier survival curves over time. The continuous divergence in survival between the two groups could be attributed to the persistent vulnerability of those who had experienced rejection within the first year, leading to more frequent and severe episodes of rejection in the subsequent years.

In essence, our findings suggest that the management of acute rejection in heart transplant recipients is not merely a short-term concern but may have long-term implications. The presence of specific risk factors, coupled with the propensity for rejection recurrence, can lead to a protracted and escalating risk of mortality over the years following transplantation. This underscores the importance of ongoing surveillance, risk assessment, and tailored therapeutic strategies for heart transplant recipients to improve their long-term outcomes.

This study must be interpreted considering limitations that should be acknowledged to provide a comprehensive understanding of its scope and potential implications. First, this is an analysis of a large observational prospective registry, and it carries the inherent limitations of this study design. Secondly, our results are based on the US organ system, and the results could be generalizable to the rest of the world only by considering the differences between different health systems. Thirdly, the study spans a considerable timeframe, from 2000 to 2021, and, over this period, there have been advancements in transplant medicine, changes in immunosuppressive therapies, and evolving clinical practices, which could have impacted rejection rates and patient outcomes without being adequately accounted for in the analysis. Then, despite rigorous data analysis, there may be additional factors influencing outcomes that were not considered in the present analysis. This includes patient-specific factors, such as genetics, socioeconomic status, lifestyle choices, and comorbidities, which could impact both the incidence of acute rejection and long-term mortality. Also, specific perioperative data, including surgical techniques and details on the use of MCS, were lacking. Finally, the accuracy and completeness of data within large registries, such as the UNOS Registry, can vary, and incomplete or inaccurate data could introduce bias or limitations in the analysis. In relation to this, it must be noticed that 10% of isolated heart transplantation patients were excluded due to missing data on rejection during the first year.

## 5. Conclusions

**Figure 4:**
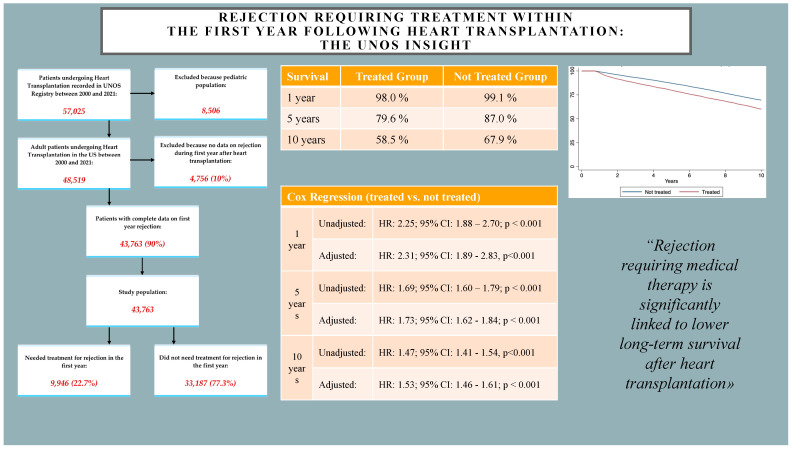
Graphical summary of the study. 95% CI: 95% confidence interval. HR: hazard ratio. UNOS: United Network for Organ Sharing.

Our study highlighted that instances of rejection requiring medical therapy are significantly linked to lower long-term survival after heart transplantation. Risk factors for early rejection should be defined and considered when making transplantation decisions and providing ongoing patient care. Further studies with better granularity of data are needed (Figure 4).

## Figures and Tables

**Table 1:** Baseline characteristics of heart transplantation recipients and donors divided into patients treated or not for rejection in the first year. BUN: blood urea nitrogen. CPRA: Calculated Panel Reactive Antibody. ECMO: extracorporeal membrane oxygenation. IABP: intra-aortic balloon pump. LVAD: left ventricular assist device. PCWP: pulmonary capillary wedge pressure. SGOT: serum glutamic-oxaloacetic transaminase. SGPT: serum glutamic-pyruvic transaminase. sPAP: systolic pulmonary artery pressure. UNOS: United Network for Organ Sharing.

Variable	No Treatment (*n* = 33,187)	Treatment (*n* = 9946)	*p*-Value
Recipients:			
Age, years	52 (±14)	49 (±14)	<0.001
Female, *n* (%)	7759 (23)	3072 (31)	<0.001
Race			
White, *n* (%)	22,765 (67)	6688 (67)	<0.001
Black, *n* (%)	6737 (20)	2127 (21)	
Others, *n* (%)	4315 (13)	1131 (12)	
New allocation system, *n* (%)	21,966 (65)	4844 (49)	<0.001
UNOS status			
1A, *n* (%)	14,632 (43)	4258 (43)	<0.001
1B, *n* (%)	8894 (26)	2934 (29)	
1 (old allocation), *n* (%)	820 (2)	170 (2)	
Prior cardiac surgery, *n* (%)	4235 (12)	2666 (27)	<0.001
LVAD, *n* (%)	4113 (12)	1069 (11)	<0.001
IABP, *n* (%)	3462 (10)	880 (9)	<0.001
ECMO, *n* (%)	710 (2)	166 (1.6)	0.007
CPRA value	11 (±23)	14 (±26)	<0.001
Creatinine, mg/dL	1.4 (±0.9)	1.3 (±0.7)	<0.001
Cardiac output, L/min	4.3 (±1.4)	4.3 (±1.4)	0.746
PCWP, mmHg	20 (±9)	20 (±9)	0.781
sPAP, mmHg	29 (±11)	29 (±11)	0.868
Donors:			
Age, years	32 (±11)	31 (±12)	<0.001
Female, *n* (%)	8840 (27%)	3124 (31%)	<0.001
History of alcohol use, *n* (%)	4972 (15%)	1059 (11%)	<0.001
Antihypertensive use, *n* (%)	9788 (29%)	2458 (25%)	<0.001
Smoker, *n* (%)	5093 (15%)	1797 (18%)	<0.001
BUN, mg/dL	21 (±18)	18 (±16)	<0.001
Creatinine, mg/dL	1.40 (±1.41)	1.35 (±1.35)	0.005
SGOT, U/L	102 (±322)	112 (±474)	0.015
SGPT, U/L	106 (±401)	106 (±388)	0.904
Ischemic time, hours	3.2 (±1.0)	3.2 (±1.1)	0.829

## Data Availability

The data presented in this study are available on request from the corresponding author. The data are not publicly available due to restrictions because the information could compromise the privacy of research participants.
